# Concerns About Theorizing, Relevance, Generalizability, and Methodology Across Two Crises in Social Psychology

**DOI:** 10.5334/irsp.1038

**Published:** 2025-05-12

**Authors:** Daniël Lakens

**Affiliations:** 1 Eindhoven University of Technology, The Netherlands

**Keywords:** history of psychology, theory, generalizability, methodology, relevance

## Abstract

During two crises in social psychology, the first from the 1960s to the end of the 1970s, and the second starting in 2010 and still ongoing, researchers discussed the strength of theories in the field, the societal relevance of research, the generalizability of effects, and problematic methodological and statistical practices. Continuing on the first part of this review, which focused on replicability, I compare similarities in the concerns raised across both crises. I consider which issues have prompted meaningful reforms and which have yet to result in significant progress. Finally, I reflect on the extent that the incentives contributed to these crises and argue that a more coordinated approach to scientific research is needed to prevent these concerns from resurfacing in a future third crisis.

‘Psychologists who are ignorant of intellectual history are condemned to repeat it in their laboratories.’

*Richard E. Nisbett, The anticreativity letters: Advice from a senior tempter to a junior tempter*

Discussions about the quality of scientific claims in social psychology – or psychology more general – are best known under the shorthand of a replication crisis. But it would be a mistake to believe that at times where researchers collectively reflected on the reliability of the scientific claims in their field were exclusively concerned with how difficult it was to successfully replicate published empirical findings. There have been two times in the history of psychology where there was a ‘crisis of confidence,’ the first from the 1960’s to the end of the 1970s, the second starting from 2010 which is to extent still ongoing today (Elms 1975; Pashler & Wagenmakers 2012), is not limited to a lack of confidence in the replicability of empirical claims, but in addition focused on the strength of psychological theories, the applicability of findings, the generalizability of results, and the adequacy and correct use of methods.

It should not be a surprise that concerns about replicability, theory, practical applicability, generalizability, and methodology are interconnected. Weak theories do not restrain analytic flexibility, making it easier to publish articles based on selectively reported tests. Lack of practical relevance means scientific claims never fail to deliver in practice, which blocks an important feedback mechanism where unreliable scientific claims are identified because they fail when implemented. If the generalizability of claims is not systematically examined, it is not possible to discover boundary conditions of theoretical predictions, and it is not possible to test auxiliary assumptions, which hinders the development of theories. Weak theories and a lack of generalizability also make it difficult to interpret failed replications. Finally, when problematic research practices such as selective reporting and publication bias are rampant, it is difficult to build theories because evidence for the accuracy of their empirical content remains too uncertain. Similarly, questions concerning the generalizability and practical relevance of claims can’t be answered despite a substantial number of studies that have been carried out. There has been an unfortunate tendency to engage in the fallacy of relative deprivation by arguing that challenges related to the replicability of findings are less important than the problems our field faces when it comes to creating strong theories, creating knowledge that has practical relevance, or concerns about the generalizability of findings. It is not a competition, and scientific knowledge generation is a complex system that requires all sub-processes to operate effectively. The fact that the same concerns have been raised during two crises should focus our attention on the properties of the system that stand in the way of successfully resolving these concerns. At the same time, it is important to point out that good science gets done. Just as during an economic crisis some companies might be highly profitable, a field can face multiple scientific crises while some research lines produce useful scientific insights. We can appreciate that some progress has been made, while acknowledging that the challenges that remain in the field at large are substantial. It would be naïve to believe we happen to live in the exact moment in history where we have achieved the best possible way to do science. Reflecting on why it has proven difficult to prevent a theory crisis, a crisis of relevance, a generalizability crisis, and concerns about methodology from resurfacing hopefully increases the awareness that we need to come together as a field if we want to resolve the underlying causes of these longstanding concerns.

## A Theory Crisis

The first crisis was strongly inspired by a general dissatisfaction with the quality of psychological theories, and a perceived lack of progress. For example, Elms (1975, p. 972) writes ‘Social psychology is clearly in need of new and better theories. Probably the most persistent complaint in the field’s history, from within and without, is that it is largely empirical, with little theoretical guidance.’ Kruglanski (1975, p. 491) expresses the point in even stronger terms, under the heading ‘Theoretical retardation’: ‘Philosophers of science concur that scientific advances are advances in theory. But the state of theory in personality and social psychology may well be reason for concern.’ The lack of theoretical progress was famously criticized by Meehl (1978, p. 807):

It is simply a sad fact that in soft psychology theories rise and decline, come and go, more as a function of baffled boredom than anything else; and the enterprise shows a disturbing absence of that cumulative character that is so impressive in disciplines like astronomy, molecular biology, and genetics.

Similar sentiments were expressed earlier by Pereboom (1971, p. 445): ‘The upshot of the problem is that theories rarely die in psychology, they just accumulate’.

Two causes of the lack of theory development are discussed in the first crisis. The first cause is an overreliance on statistical and methodological developments, and too little thought about the theoretical questions that are being asked. Cartwright (1979, p. 87) notes:

But these impressive gains in technical competence and sophistication have been, I fear, something of a mixed blessing, for the fascination with technique seems all too often to have replaced a concern for substantive significance. The literature is full of studies that do little more than demonstrate the technical virtuosity of the investigator, and one might think that our journals would have to go out of business if use of the analysis of variance were to be prohibited.

Similarly,

Another impression given by these multiple exercises in partial theoretical integration is that in the building of our science, we over-value *p*-levels and undervalue the judgmental appraisal of evidence. The bricks may be culled by *p*-level, but the mortar, the girders, the theoretical structure are necessarily judgmental through and through, with little help from statistics (Smith, 1972, p. 94).

This concern still plays a role during the second crisis, as for example expressed by Gigerenzer (2018, p. 17): ‘Second, and most important, obtaining statistical significance has become a surrogate for good scientific practice, pushing principles such as formulating precise theories, conducting double-blind experiments, minimizing measurement error, and replicating findings into the sidelines.’ In the first crisis psychology was seen as an accumulation of facts which were not integrated into theories:

The respect of common sense, the proliferation of experimental studies lacking theoretical preoccupations, and the isolation of various areas of research in social psychology combine to explain the accumulation of facts and notions which do not amount to real progress since they are not conceptually integrated and since no theory is, in any real sense, disconfirmed or replaced by another (Moscovici, 1972).

When it comes to the quality of theories in psychology, there seems to have been little progress in general. In a 2004 special issue that had the goal to strengthen training in theory construction in social and personality psychology, Kruglanski and Higgins (2004, p. 96) write ‘Theory construction plays an essential role in any field of science. We believe that social personality psychology has tended over time to be more phenomenon and data driven than theory driven and that this state of affairs may have impeded scientific progress in our field’. The continued criticism on theorizing in social psychology was repeated during the second crisis: ‘we argue that the crisis runs much deeper and is ultimately rooted in theory or lack thereof. Many subfields within psychology (though not all!) lack any overarching, integrative general theoretical framework that would allow researchers to derive specific predictions from more general premises’ (Muthukrishna & Henrich 2019, p. 221). Similarly, Oberauer and Lewandowsky (2019, p. 1596) write:

Here, we argue that, in addition to poor methods, the replication crisis is also due to the prevalence of theories that have only a weak logical relation to the hypotheses through which they are evaluated empirically. We suggest that the replication crisis is best resolved by focusing attention on the role of theorizing, and we do not believe that current recommendations that focus entirely on data generation are sufficient to overcome the crisis.

In addition to a replication crisis, researchers speak of a theory crisis (Eronen & Bringmann 2021). Proposing new theories is – if anything – rewarded too much. Mischel (2008) refers to this as the ‘toothbrush problem’ where researchers prefer to propose their own theory, instead of building on the theory of someone else. The main problem in the theory crisis is that theories are vague (Frankenhuis et al. 2023). Psychological theories too rarely reach a point where they are well-specified, consistently applied across papers, and formalized.

Researchers discuss how the reward structures in psychology favor ‘fun’ studies over studies that make a theoretical contribution. The observations of Ring (1967, p. 117) during the first crisis are worth citing in full:

Clever experimentation on exotic topics with a zany manipulation seems to be the guaranteed formula for success which, in turn, appears to be defined as being able to effect a tour de force. One sometimes gets the impression that an ever-growing coterie of social psychologists is playing (largely for one another’s benefit) a game of ‘can you top this?’ Whoever can conduct the most contrived, flamboyant, and mirth-producing experiments receives the highest score on the kudometer

and

The implicit values that produce this sort of research include the following: 1. Experiments should be as flashy and flamboyant as possible. 2. If you can think of an effective manipulation, fine; if you can think of an effective manipulation that is also amusing, even better. 3. If the topic selected for study is itself prosaic, you should reconsider; if you go ahead, at least study it cleverly. 4. Never make an obvious prediction.

Similarly, Pereboom (1971, p. 449) writes ‘In the light of all the previous discussion and the evidence, it no longer seems productive to continue to invent “cute” experiments or increase the sophistication of our experimental designs while decreasing the breadth of our theories’.

These concerns are echoed in the second crisis by Ledgerwood and Sherman (2012, p. 63): ‘Placing a premium on brief reports of flashy findings can exacerbate the issue of declining effects, as single-study findings that lack a theoretical context or that contradict previous data are especially likely to be spurious.’ Similarly, Giner-Sorolla (2019, p. 14) explicitly references the work by Ring from the first crisis when stating ‘Social psychologists with very different opinions on the crisis of evidence have voiced concerns about publishing attention-grabbing, media-friendly research with surprising or “sexy” results – recapitulating Ring’s (1967) concerns about “fun-and games” “social psychology” at the onset of the previous crisis.’ There are some preliminary meta-scientific indications that media-friendly research is less replicable (Youyou et al. 2023). Although some journals in psychology, such as the journal of Personality and Social Psychology, managed to convince researchers to submit multi-study scientific articles in the past, the replication crisis followed a period in which there was an increasing number of scientific journals that introduced short report publication formats.

The fact that almost identical concerns about the state of psychological theorizing are raised in the first and the second crisis is a source of concern, especially given the lack of practical reform that aims to improve good theorizing. Where the replication crisis has led to notable changes in the number of replication studies that are performed and published, journal policies about publishing close replication studies, funding for replication research, reflections on which studies are worthwhile to replicate, the transparent reporting of statistical analyses, and discussions about how to resolve ambiguities about diverging results across close replication studies, there are as of yet no similar concrete improvements implemented that can be expected to improve theorizing in psychology. Concrete suggestions to improve theory development in psychology deserve much greater attention. Some first steps have been made (Borsboom et al. 2021; Glöckner & Fiedler 2024; Leising et al. 2023; Maier et al. 2024), but it will remain a challenge to improve this longstanding problem in practice.

Borsboom and colleagues point out three reasons why there continues to be so little progress in improving theorizing in psychology. They write:

psychologists (a) lack a collective, coordinated research program on theory formation; (b) are rarely trained to develop skills conducive to theory development; and (c) live in a research culture that endorses the norm that science is defined by its methods of hypothesis testing rather than theory construction more broadly. (Borsboom et al. 2021, p. 757).

These first two themes – a lack of coordination, and a lack of training – are also important reasons for why there has been little change in other issues raised during both crises, such as the crisis of relevance and the generalizability crisis. The lack of specialization in psychology is in part due to the fact that researchers are rewarded based on their individual performance and not based on the performance of a collective (Tiokhin et al. 2023). If researchers coordinated their efforts, some individuals could focus on developing expertise in theory development and formalization. As Borsboom and colleagues point out, expertise in theory development and formalization is currently not rewarded as much as expertise in research methods, and therefore there are not enough expert theoreticians to help their peers raise the level of theory development in psychological science.

## A Crisis of Applicability

During the previous crisis some of the most eminent psychologists publicly doubted the societal impact that psychologists have. In a presidential address to the American Psychological Association George Miller (1969, p. 1063) stated:

As a science directly concerned with behavioral and social processes, psychology might be expected to provide intellectual leadership in the search for new and better personal and social arrangements. In fact, however, we psychologists have contributed relatively little of real importance —even less than our rather modest understanding of behavior might justify.

In part, this criticism was a consequence of the increased reliance on lab-based experiments (as opposed to more naturalistic paradigms or field studies), as illustrated by Deutsch (1976, p. 136): ‘current theory is deficient in characterizing both the socially relevant properties of individual personalities and the psychologically relevant attributes of social situations.’ The criticism of the lack of societal impact that psychology had was surprisingly widespread during the first crisis. Helmreich (1975, p. 558) writes ‘However, several outcomes do seem possible. One is that the pursuit of social psychological truths will degenerate into a form of laboratory-based, mental masturbation, valid in its own right, but devoid of contact with mundane reality.’ Doing more applied research is proposed as a solution to the problems in psychology by Pereboom (1971, p. 451): ‘In applied research our objectives can often be made very explicit. This in itself solves many problems.’

At the same time, it is observed that psychology needs to improve to generate knowledge that will be applicable. Aron (1979, p. 50) notes:

Even though we have discovered quite a bit in the limited areas we have been able to research, what we have found has not been successful in application. It is important to note, however, that this is not because the principles we found were wrong — when they have been applied they work. Rather, the difficulty has been that the kinds of laws we have found through our experiments, laws governing the social influences on individuals, either cannot or will not be applied by society.

One reason for the lack of more relevant research is dominance of ‘hit-and-run’ studies (Kruglanski 1975). In the provocatively titled article ‘Why social psychology fails’ Silverman (1977, p. 355) notes:

First, we note that the experiment, defined in the classic sense of manipulation of the independent variable, remains the method of choice. This would not necessarily be disquieting, except as it relates to a second observation, that social psychologists in the field continue to work in the pattern of heterogeneous, short-term studies, of very limited impact on subjects, that predominated in the laboratory.

Psychologists continued to raise concerns about how lab-based studies remained too far removed from actual behavior to make psychology practically applicable (e.g., Baumeister et al. 2007), the lack of recognition of the importance of field studies (Cialdini 2009), or how psychological findings are often not ready to inform policy (IJzerman et al. 2020), although there have also been defenses of the practical relevance of psychological research (Lilienfeld 2012; Zimbardo 2004). Nevertheless, during the second crisis the relevance of the field for societal wellbeing was rarely at the center of attention. Two exceptions come from Giner-Sorolla (2019), who reviews concerns on the relevance of the questions psychologists ask, the generalizability of findings due to the samples that are collected, and the way psychological science is communicated to the public, and Berkman and Wilson (2021, p. 4), who note: ‘Our casual observation is that psychological theory has become unmoored from the guiding principle of practicality and is drifting toward more nuanced or myopic theoretical questions that are less relevant to helping solve the problems that people care about’. During the second crisis, doing more applied research is seen as a possible solution to concerns about generalizability (Yarkoni 2021).

It is surprising that the societal relevance of psychological science is not a topic of discussion in the second crisis, as not much has changed with respect to the research that is done. In response to the concerns raised by Ring (1967) about the prevalence of flashy experiments, McGuire (1967, p. 126) predicted the following: ‘I foresee that the ingenuity now exercised in creating laboratory surrogates for the real world will be steadily replaced by equal ingenuity exerted in utilizing the natural environment as a field in which to test these deductions.’ Early meta-scientific studies examining the prevalence of field studies showed there was no increase between 1961 and 1970 (Fried et al. 1973; Higbee & Wells 1972). The situation has not noticeably improved in the years since, and the hope that psychologists would perform more field studies in the future has not materialized (Doliński 2018). In general, researchers today are still concerned about the pressure to publish small but flashy studies: ‘Currently, swiftly publishing several small but catchy contributions trumps publishing a carefully planned multi-study investigation, because institutional incentives require boosting one’s publication count to excel in short-sighted performance metrics.’ (Rahal et al. 2023, p. 165).

Although published articles where researchers express their doubts about the relevance of psychological science are largely absent during the second crisis, I doubt these concerns have disappeared. Instead, given that this topic regularly comes up in conversations I have with fellow academics, the concern might still exist but is no longer discussed openly. Perhaps we have become less comfortable expressing our doubts about the relevance of our field publicly, but the topic is important enough to attempt to bring it more in the open for those researchers who are concerned about the societal relevance of our field. Applied research in general, and field studies specifically, take a lot of effort to do well. Researchers often need to build a network that makes it possible to perform the studies, which requires extensive time investment. Most researchers, and especially those on short-term contracts, do not have the resources to perform the research required to demonstrate the practical relevance of psychological science. Results from applied research are often less conclusive than lab-based studies, and applied research is on average less novel than theoretical studies, which reduces their value in the current reward structure in academia. If psychologists want to contribute more of real importance, as Miller (1969) urged them to, they will need to come together and re-organize their science around more collaborative and coordinated research efforts where practical relevance is prioritized as a shared goal, and resources are combined to pursuit this goal.

## A Generalizability Crisis

Related to concerns about the relevance of psychological research for society, researchers expressed a concern during the first crisis that psychological findings failed to generalize. Silverman (Silverman 1971, p. 583) noted:

If the multitude of social-psychological findings cannot aid the planners of society, it is apparently not because we have been researching the wrong topics. It must be that our data are not generalizable to the objects of our studies in their natural, ongoing states. This is a basic inadequacy of methodology rather than direction, and it will not be resolved by pontifical edicts from any source about what to study and where.

Similarly, Epstein (1980, p. 790) writes: ‘in the event that a result is replicable, there is little likelihood that it will be sufficiently general across minor variations in stimulus conditions to identify scientifically useful relationships.’ One concern about the lack of generalizability of psychological science was a direct consequence of Gergen’s view that psychology should be seen as history. He argued that psychology deals with findings that do not remain stable over time, which implies that effects are not repeatable. It should be noted that this idea, although popular among some, was also strongly criticized at the time, with for example Schlenker (1974, p. 2) stating ‘Gergen’s arguments typify several popular misconceptions about the nature of science and exhibit some undue pessimism’.

Another concern during the first crisis is the lack of representativeness in participants (Carlson 1971; Triandis 1975). For example, McGuire (1967, p. 313) writes: ‘Probably the most important of the difficulties is the growing realization among laboratory experimenters that our work is troubled by artifacts that make generalization and theoretical interpretation difficult.’ He continues: ‘We have almost grown used to the embarrassment occasioned by the concentration of our research on college students. The proverbial ‘psychology of the college sophomore’ is more worrisome as to generalizability for the social psychologist than for those working in perception, learning, etc.’ Schultz (1969) agrees: ‘In reading our journals, one receives the distinct impression that the only kind of people of interest to psychologists are college students! If college students were truly representative samples of the population at large, there would be no problem in generalizing from the results of our studies. But (fortunately or unfortunately) they do differ in highly significant ways from the general population, and we cannot have a truly meaningful science of human behavior by studying such a restricted sample.’

Participants in field studies are not necessarily more diverse. A meta-scientific study by Dipboye and Flanagan (1979) found that both field studies and lab experiments sample from a limited range of subjects, settings, and behaviors, and that both types of studies therefore provide little guarantees that observed findings will generalize. Helmreich (1975, p. 551) raises the additional concern that it is difficult to generalize from the acute responses in the laboratory to effects on a longer timescale: ‘Subjects are usually held for at most one or two hours, leaving the researcher forced to generalize from a very short time frame to trends of interaction and response over months or years.’ Some came to the defense of artificial lab-based experiments (a notable example is Mook’s (1983) article ‘In defense of external invalidity’), but the lack of generalizability of research findings in psychology remained a concern, especially among those psychologists who also lamented the lack of practical relevance of the field.

The same concerns have been raised during the second crisis by Yarkoni (2021, p. 12): ‘At the same time, the current focus on reproducibility and replicability risks distracting us from more important, and logically antecedent, concerns about generalizability. The root problem is that when the manifestation of a phenomenon is highly variable across potential measurement contexts, it simply does not matter very much whether any single realization is replicable or not’. There have been some exciting developments in the second crisis to address concerns about generalizability, and perhaps not surprisingly, these developments have mainly consisted of large collaborative projects. The Psychological Science Accelerator (Moshontz et al. 2018) brings together scientists who have the shared goal to accumulate reliable and generalizable evidence in psychological science by performing research in a wide range of different countries. Awareness has increased about the large heterogeneity in effect sizes within research areas (Linden & Hönekopp 2021), and researchers have begun to explore this heterogeneity more systematically in experiments where factors that might limit the generalizability of findings are systematically explored (Almaatouq et al. 2022; Baribault et al. 2018). Similarly, large-scale experiments have been used to systematically examine and explain heterogeneity in effect sizes with the goal to improve theory building (Krefeld-Schwalb et al. 2024). Although it is too early to evaluate the success of these approaches, these projects provide initial indicators that concerns about generalizability are being taken more seriously. Exploring the generalizability of findings will most often require substantially more resources than examining an effect in a single context. Given the limited resources most individual researchers are faced with, systematically examining generalizability will require a coordinated approach.

## A Methodological Crisis

Concerns about methodological and statistical practices that inflate the probability of observing a false positive (or Type 1 error) are centuries old (Babbage 1830). Neher (1967) coined the term ‘probability pyramiding’ for the inflated Type 1 error rate due to the practice of researchers to perform multiple tests, and the combined effects of Type 1 error inflation during the data analysis with the practice to selectively write up studies with significant findings, and publication bias favoring statistically significant results. Foreshadowing the well-known article ‘Why most published research findings are false’ by Ioannidis (2005), Neher wrote: ‘By seemingly conservative estimates, the probability that a finding (reported at *p* = .05) is a result of chance is not .05 but is closer to .50. That is, were these estimates approximately correct, they would indicate that about one-half of the original findings reported at this level in behavioral science journals could have resulted solely from chance variations.’

In 1976 Barber published the book ‘Pitfalls in Human Research: Ten Pivotal Points.’ In chapter 4 on ‘Investigator Data Analysis Effect’ he presents nine potential issues: 1) performing unplanned analyses, 2) hypothesizing after results are known (cf. Kerr 1998), 3) performing a large number of tests and only reporting significant results, 4) cutting and slicing data in originally unintended ways, 5) not correcting for multiple comparisons, 6) selectively reporting significant results, 7) relatedly, not reporting non-significant results, 8) checking for errors after negative results, but not checking for errors after positive results, and 9) reporting statistically significant but practically insignificant effects (Barber 1976). Several of these issues are remarkably similar to ‘researcher degrees of freedom’ presented in the seminal article on false positive psychology that played an important role in increasing awareness of research practices that inflate the probability of false positive results during the second crisis (Simmons et al. 2011).

Even though data analysis practices that inflate the Type 1 error rate were known, there was basically no discussion of these problematic research practices during the first crisis. More general methodological criticisms were important in the first crisis (Faye 2012; Higbee & Wells 1972; McGuire 1973), but the focus was on how demand effects and experimenter effects introduced confounds in lab experiments (Orne 1962; Rosenthal 1966).^1^ The realization that thoughts and feelings of both participants and experimenters could introduce systematic bias was discussed extensively throughout psychology’s history (for a historical review, see Morawski 2015), but new empirical demonstrations were a real shock to social psychologists in the first crisis (Argyris 1968; Schultz 1969), and increased the criticism on lab-based studies.

At the same time, the first crisis largely ignored research practices that inflate the Type 1 error rate. We find a hint of a recognition of the problem in M B Smith (1972, p. 94) when discussing how *p* values are not as tightly linked to support for our theories as we typically pretend, because researchers and editors selectively report positive findings: ‘We also know about experimenters’ practices in the use of pretests and pilot runs to decide whether a manipulation is appropriate-and about editors’ lack of interest in publishing negative results.’ But it is fair to say that these bad research practices were not publicly discussed. This is regrettable, as an honest discussion of these problematic practices during the first crisis could have prevented considerable research waste. For example, better research practices might have prevented a research literature of 198 tests of the ego-depletion effect from 83 experimental studies that seemed to show a substantial and reliable effect (Hagger et al. 2010), when later preregistered replication studies involving many of the same authors found no effect despite a much larger sample size (Vohs et al. 2021). A systematic meta-analytic review of the mortality salience literature included 825 studies, but due to overwhelming evidence of bias, it remained uncertain whether there was a mortality salience effect to begin with (Chen et al. 2024). As a scientific discipline we should be deeply concerned with the fact that peers can perform hundreds of studies without generating any reliable knowledge. Although it is difficult to quantify how much research efforts were wasted, both because we do not have access to all research that was performed (Lakens & Ensinck 2024), and because we do not know which research lines are completely built on Type 1 errors, there can be little doubt that questionable research practices must have led to research waste.

The second crisis started with the realization that commonly used research practices allowed researchers to make scientific claims that turned out to be less reliable than researchers realized. The publication of an article by Daryl Bem describing nine studies that provided empirical support for extra-sensory perception (Bem 2011), as well as the creation of open access journals that published failed replication studies of prominent findings in the field (Doyen et al. 2012) started a widespread discussion about problematic methodological practices (LeBel & Peters 2011; Wagenmakers et al., 2011). Accessible articles that explained how problematic research practices increased the Type 1 error rate (Simmons et al. 2011), together with surveys suggesting many researchers admitted to using these practices (John et al. 2012) caused at least as great a shock among psychologists as the realization that participants and experimenters had thoughts and feelings had in the first crisis. The fact that these issues seem obvious in hindsight, and yet persisted for decades, is a good reason to display intellectual humility. After all, who knows which practice that we currently engage in will be at the root of the next crisis in psychology?

Especially early on in the second crisis accusations about questionable methodological practices co-occurred with highly visible discoveries of scientific fraud, such as the case of Diederik Stapel (Sijtsma 2023). For a long time, it remained unclear whether and how data manipulation and *p*-hacking could be distinguished. For example, an investigation of the probability that sets of published findings would have yielded exclusively positive results, Francis (Francis 2014, p. 1183) considered the practice to intentionally publish only positive results ‘essentially fraud.’ Dirk Smeesters, who resigned after an investigation into data manipulation, defended himself by arguing others in their field used similar practices (Enserink 2012). The strong uncertainty about which methodological practices could now lead to an accusation of data fraud strongly motivated researchers to signal they did not engage in practices that violated research integrity. Researchers quickly embraced practices to distinguish exploratory from confirmatory tests (Wagenmakers et al. 2011). The development of the Open Science Framework allowed researchers to preregister their research, and the first published study using the Open Science Framework was accepted for publication in March 2013 (LeBel & Campbell 2013). Preregistration improves the ability of peers to evaluate how severely hypotheses have been tested (Lakens 2019), even though in practice preregistrations are often not implemented according to best practices (Van den Akker et al. 2023).

The increase in awareness of the problems associated with selective reporting and Type 1 error inflation has been substantial. It is too early to evaluate if there will be lasting effects on research practices in psychology, and it will be practically impossible to make causal claims (Lakens et al. 2024), but it seems plausible that the second crisis has changed methodological practices in psychology. Beyond psychology, the second crisis has instigated an interest in improving research practices in other scientific disciplines, such as ecology (Fraser et al. 2018), sports science (Mesquida et al. 2023), and economics (Brodeur et al. 2016). Researchers working on better methodological practices have been relatively successful compared to researchers who have tried to improve theory building, applicability of psychological research, or generalizability. A possible reason for this success is that researchers have the ability and resources to change their own practices, and peer reviewers have a direct influence on researchers, and can ask them to address bad research methods. Improving psychological methods is perhaps not easy, and there is still a lot to be done, but as methods and statistics are mostly within the control of individual researchers, these aspects of the research process are easier to improve.

## Are Crises Caused by Bad Incentives?

The belief that the incentive system in academia puts pressure on researchers to prioritize quantity over quality is at least a century old. Case (1927, p. 325) wrote: ‘If it be true that, for the time being at least, the quality of American sociological writing is in inverse ratio to its quantity, the reason is to be sought, among other things, in the fact, first, that the system of promotion used in our universities amounts to the warning, ‘Publish or perish!’.’ Elms discusses how pressures from outside academia to do relevant research, the realization of biases after the emancipation of women and racial minorities, and concerns about ethical standards in the field as a cause of the feeling of a crisis. He remarks (1975, p. 972): ‘The contributions that other factors have made to the crisis of confidence are less clear: for instance, the publish-or-perish pressures and shrinking job market within the academic world’. Still, he also believes that ‘social psychologists must find ways of adjusting to or moderating academic pressures to publish, at the same time that they reduce their own rate of publishing findings that are so incomplete as to be misleading.’ Sherif is clearer on the negative effect of the incentive system on the quality of research (1977, p. 371): ‘Even from this quick glance at the state of two major areas of research, a rather clear but somewhat disturbing picture stands out; piles and piles of output in research and publication, accelerated almost daily by the ‘publish or perish’ policy of academia. In brief, the ratio of golden kernels to be saved to the huge quantity of chaff is only a minute fraction.’ Similarly, Mahoney (1979, p. 357) notes: ‘Of course, this tendency to publish (and speculate) quickly is reinforced by the reward system in science, which gives strong encouragement to volume, as well as to velocity. Studies of the academic scientist leave little doubt that ‘publish or perish’ is more than a glib alliteration.’

One bad incentive that deserves particular attention is the tendency of psychological scientists to prefer positive results over negative results, and publish the former, and fail to publish the latter. In a section ‘The Secrets We Keep’ Dunnette writes:

We might better label this game “Dear God, Please Don’t Tell Anyone.” As the name implies, it incorporates all the things we do to accomplish the aim of looking better in public than we really are. The most common variant is, of course, the tendency to bury negative results. I only recently became aware of the massive size of this great graveyard for dead studies when a colleague expressed gratification that only a third of his studies “turned out” – as he put it.

Greenwald performed a meta-scientific study surveying reviewers and authors of articles submitted to the Journal of Personality and Social Psychology to provide the first model and parameter estimates of the causes of publication bias. In the second crisis we have seen a widespread interest in the development and application of statistical methods to identify bias in the scientific literature (Francis 2014; Schimmack 2012). Where some fields, such as clinical trial research, have created regulations that require all trials to be registered and updated with the results (Dickersin & Rennie 2012), no systematic solution for publication bias has been implemented in psychological science. Given that positive results continue to be valued more than negative results, more action will be needed to prevent the negative effects due to publication bias on the generation of scientific knowledge in psychology (Lakens & Ensinck 2024).

In my view, it is the lack of a coordinated approach to knowledge generation that is the underlying cause of most of the problems raised in both crises (Rasti et al. 2025b). Because most psychologists work in small teams, often consisting of only one or two supervisors and PhD students, or at most a handful of collaborators, they are limited in the expertise they have access to, and the scope of the challenges they can tackle. Adequately testing competing theories requires a massive amount of work, from conceptualization, building theoretical models, measurement development and validation, pilot tests to examine auxiliary hypotheses, exploratory research to derive predictions, and tests of hypotheses and alternative explanations. Small teams are now responsible for all aspects of these steps, and the level of expertise that can be attained in all these necessary aspects of empirical research is therefore limited. Furthermore, each step in the theory testing process depends on previous steps, and these interdependencies require some amount of coordination to occur efficiently.

To really resolve problems related to replicability, methodology, generalizability, theory, and relevance, researchers need to be embedded in an overarching research collaboration where they are committed to shared goals, such as developing valid and reliable measures, establishing replicable effects, examine their generalizability and applicability, and severely test competing theories. For such a collaboration to be successful, it requires some form of management and the ability to create a long-term planning, so that interdependent research projects can be completed effectively (Rasti et al. 2025a). Currently, psychologists work in a system best described by Polanyi (1962, p. 56) where scientists are producers and consumers of knowledge in a marketplace, where they are driven by an ‘invisible hand’ to ‘to produce the highest possible result by the use of a limited stock of intellectual and material resources.’ In essence, I believe the problems raised in both crises constitute a form of market failure. Without some form of coordination and long-term planning, many of the challenges identified in the two crises cannot be resolved. Even a psychologist who ignores the incentives in their field will be limited in the contributions they can make to the challenges concerning replicability, theory, applicability, generalizability, and methodology. Whether it is the difficulty of drawing conclusions about Schachter’s cognition-arousal theory of emotion after two decades of research during the first crisis (Reisenzein 1983), or the difficulty of drawing conclusions about the relationship between videogames and aggression during the second crisis (Ferguson et al. 2020), theoretical progress in psychology is frustratingly slow, if there is progress at all on some research questions. Although current academic incentives too often hinder progress, even without these negative external influences the challenges that psychologists face are simply too substantial to resolve without a change to the system in which psychologists work.

The incentives in academia are often mentioned in articles written during the second crisis (e.g., Chambers 2019; Fraser et al. 2018; Rahal et al. 2023; Sarafoglou et al. 2022; Schimmack 2012). It is important to point out that criticism of the incentives in academia is not limited to the pressure to publish, but also encompasses other aspects of academic life, such as employment conditions of early career researchers that make it impossible to make long-term plans, or a high workload that prevents researchers from having sufficient time to educate themselves. What’s new is that the incentive structure is not just presented as a cause of some of the problems that the discipline faces (Fiedler et al. 2012; Giner-Sorolla 2019; Heesen 2018; Koole & Lakens 2012; Romero 2019), but also as a malleable aspect of the scientific ecosystem that can be changed (e.g., Maner 2014; Nosek et al. 2012; Schimmack 2012; Tackett et al. 2017; Tiokhin et al. 2023). Meta-scientists have started to experiment with a range of possible interventions to reward good research practices, such as the Registered Report publication format (Chambers et al. 2014; Nosek & Lakens 2014), and peer reviewers who commit to only review scientific articles that either share data or explain why data cannot be shared (Morey et al. 2016). Some interventions, such as badges that identify articles that are preregistered or have open data, have already been discontinued in some journals (Hardwicke & Vazire 2023). Perhaps due to the greater number and diversity of scientific journals, there is a lot more experimentation in journal policies and publication formats in the second crisis than during the first. Not all of these interventions that aim to change the reward structures in science can be expected to be successful, and some might even be counterproductive. It is often too early to be able to empirically evaluate the consequences of these interventions, but initial meta-scientific research suggests some novel developments have achieved their aim. For example, Registered Reports allow more non-significant results to be published, while the articles are at least as high-quality as standard publications (Allen & Mehler 2019; Scheel et al. 2021; Soderberg et al. 2021).

## Discussion

After two crises, what has changed? With respect to methodological practices the first crisis made psychologists more acutely aware of demand effects, although the interest initially decreased, and later reemerged (Sharpe & Whelton 2016), reminding us that there are no guarantees any changes that occur during the second crisis will last. With the increase of computer-based experimental paradigms, and a move away from extensive deception-based experiments, the influence of experimenter effects that played a central role in the first crisis (Rosenthal 1966) might be less of an issue in the second crisis (O. Klein et al. 2012), but demand effects continue to play a role (Corneille & Lush 2023). The second crisis created widespread awareness of the importance of replication research, and research practices that lead to unreliable statistical inferences, the importance of Type 1 and Type 2 error control, and the detrimental effects of publication bias.

With respect to generalizability the first crisis increased interest in the social constructionist movement (Gergen 1985). Even if it might not have become the dominant approach in psychology (Stroebe & Kruglanski 1989), it forced mainstream social psychologists to acknowledge the inherent context-dependency of their field, up to the point that if you can today summarize all knowledge generated by social psychology in two words: ‘It depends.’ At the same time, there is room for improvement. Researchers still tend to overgeneralize in the claims they make (Simons et al. 2017; Yarkoni 2021), but steps have been made to develop ideas about how to design studies that allow for more generalizable conclusions (Almaatouq et al. 2022; Baribault et al. 2018).

The second crisis led to a strong expansion of the burgeoning field of meta-science. Faust (2005) credits Paul Meehl and himself for the invention of meta-science, and defines the field as follows: ‘Meta-science, in essence, involves the application of more rigorous methods than are presently used in the history and philosophy of science to answer questions about how science does and does not work best.’ Of course, meta-science has a much longer history, but it has seen an immense increase on popularity since the second crisis, establishing itself as its own field, with dedicated scientific journals (Carlsson et al. 2017; Simons 2018). Both crises have provided fertile grounds for new scientific approaches and research areas.

Doubts about the relevance of psychological science have largely disappeared from public discourse, and do not seem to be a central concern in the second crisis in psychology. The idea that the incentives will need to change to increase the relevance of psychological science (Berkman & Wilson 2021; Giner-Sorolla 2019) has gone unchallenged. The recent explosion of research into how social and behavioral science could contribute to the COVID-19 response provided a good testcase of the relevance of psychological research for society (Bavel et al. 2020). Concerns about the lack of generalizability of psychological findings were raised (Bryan et al. 2021; IJzerman et al. 2020) and mirror the concerns about generalizability that were so important in the first crisis. An evaluation of whether claims made at the start of the pandemic could benefit society shows many claims lack any evidence for observable outcomes, and effect sizes are often small (Ruggeri et al. 2024). This does not necessarily mean the claims were not correct, but it suggests that the evidence for practical impact is scarce. Where a well-coordinated science might have used the pandemic to demonstrate its usefulness to the general public, psychologists did not manage to do so. Many of the claims based on psychological research that were best supported during the pandemic are also rather trivial (e.g., for public health messages to be effective, they should be shared by trusted sources). Overall, there seems to have been very little that psychologists have contributed to reducing the duration or impact of the pandemic that a member of the general public would have noticed and have been grateful for. This makes the absence of a discussion about the relevance of psychological science during the second crisis even more surprising. Perhaps the current generation of psychological scientists value practical relevance less compared to the psychologists active during the first crisis. It is also possible that concerns about the relevance of psychological science are still present but are simply not publicly discussed, for example because the researchers who would voice these concerns choose to leave academia (Kis et al. 2022). This topic deserves further attention, as concerns about the relevance of the field also remained unresolved during the first crisis. Just as how the absence of an honest discussion about inflated Type 1 error rates during the first crisis came back to haunt psychologists in the second crisis, lack of an honest discussion concerning the societal relevance of psychology during the second crisis might come back to haunt the field in a third crisis.

With respect to theory there has arguably been little progress, despite repeated reminders that psychologists should improve their theorizing. There have been no notable changes in journal policies to promote better theorizing during the second crisis, and there is a lack of meta-scientific work on what makes theories in the literature more successful, and what makes them less successful. Meehl’s work on cliometric metatheory (Meehl 1992; Meehl 2002; Meehl 2004) offers an exciting and, as of yet, unexplored approach for meta-scientists interested in improving theorizing in psychological science. The theory crisis is not the only fundamental issue that has remained unresolved after both crises, as there are at least equally fundamental issues concerning measurement and validity that have received very little attention in the first crisis, and far too little attention in the second crisis (Flake et al. 2017; Schimmack 2021b; Vazire et al. 2022). Concerns about the validity and reliability of specific measures have been raised, such as in the case of the competitive reaction time task (Elson et al. 2014), and the implicit association test (Schimmack 2021a), and recently more general concerns about the fragmented nature of psychological measures have been raised (Anvari et al. 2024). Nevertheless, I would not be surprised if issues related to measurement and validity have to wait until the third crisis in psychology before they receive the attention they deserve.

With respect to concerns about the incentive structure in academia, only minor changes were introduced during the first crisis. Examples are requiring authors to report methodological procedures in more detail to enable direct replications (Greenwald 1976), and the launch of the Journal of Applied Social Psychology to stimulate research with more direct relevance for society (Helmreich 1975). Much more substantial changes to the reward structure have been introduced during the second crisis, although it is too early to tell if these will prove effective. The second crisis has coincided with rapid technological advances that have made greater transparency possible and has led to the emergence of the Open Science movement. The internet has also facilitated team science, which has enabled large scale replication projects (Klein et al. 2014; OSC 2015), more representative data collection from laboratories across the world with the goal to improve generalizability (Moshontz et al. 2018), and adversarial collaborations in which theoretical predictions are sharpened and tested (Coles et al. 2022). These developments are still the exception rather than the rule. The future will tell whether these changes to the way psychologists work will become more popular and realize their potential to improve psychological science.

While for some researchers the fundamental problems that were discussed during the first and second crisis deserve all the attention they have received, others are concerned and annoyed when their discipline is faced with a widespread crisis narrative. In a footnote, Zajonc reflects on the first crisis (1989, p. 347):

Some expressions of epistemological malaise have been outright destructive and paralysing for social psychology. Entirely without heuristic consequence has been the argument about ‘social psychology as history’ […]. Nevertheless, the controversy has discouraged promising students from entering the field and granting agencies from increasing social psychological research budgets.

Similar concerns for negative effects of a crisis narrative have been expressed during the second crisis by Baumeister (2016, p. 156):

Another set of potential winners are researchers in nearby fields who compete with social psychologists for grant funds, awards, faculty lines, graduate student support, and pages in broad-readership journals. As social psychologists persist in discrediting our field’s work, we lend ammunition to other areas who argue that the precious and scarce research funds should be diverted away from social psychology and into their own areas.

Although these concerns are understandable, sometimes events happen that fundamentally challenge a scientific discipline, and the concerns that emerge are too widespread to prevent a temporary negative effect on the reputation of a discipline. Of course, the incentive structure equally exerts its pressure on those who claim there is a crisis, as Sherif (1977, p. 372) already observed: ‘There is the danger of further accumulation of a whole big crop of crisis literature because it is attention getting; it is likely to insure its author handsome professional mileage.’ Yet, such skepticism seems unwarranted. There has been too much agreement throughout and across the two crises about the ways in which psychological science can be improved to dismiss these concerns as careerism. In a conservative system such as science it might require a crisis to instigate self-reflection on how a field can improve (McGuire 1973; Mills 1979), and to motivate scientists to embrace change.

## Footnotes

This is especially ironic given that Barber and Silver raised the criticism that the evidence of experimenter effects itself suffered from inflated Type 1 error rates due to flexibility in the data analysis.
